# Alcohol and illicit drug use among potential HIV vaccine efficacy trial volunteers along Lake Victoria, Uganda

**DOI:** 10.1186/1742-4690-9-S2-P219

**Published:** 2012-09-13

**Authors:** I Ssekandi, A Ssetaala, J Mpendo, A Nanvubya, L Nielsen, N Kiwanuka

**Affiliations:** 1 UVRI-IAVI HIV Vaccine Program, Kampala, Uganda; 2 International AIDS Vaccine Initiative, New York, USA; 3 Makerere College of Health Sciences School of Public Health, Kampala, Uganda

## Background

HIV has adversely affected fishing communities (FC), with prevalence ranging between 25-30% on Lake Victoria. To better characterize the HIV prevalence among FC and explore potential for HIV vaccine efficacy trials, we conducted a pilot study on HIV prevalence, sexual risk behavior, alcohol and drug use in these communities.

## Methods

A cross-sectional HIV prevalence survey was conducted in 8 FC over 3 months. Census and mapping to determine the average population size in each community were conducted prior to the study. After obtaining informed consent, 2,200 individuals aged 18-49 years from randomly selected households were tested for HIV and responded to questionnaires on sexual behavior, alcohol consumption and drug use.

## Results

Fifty three percent (53.0%) had consumed alcohol in the past three months with males consuming more alcohol than females [59.0% vs. 46.8%, p<0.01].19.2% of alcohol consumers also used illicit drugs [p<0.01]. Alcohol consumption in the previous 3 months was highest among Catholics [p<0.01], those with no formal education [p=0.01], sex workers (100%), loaders/off loaders (79%), bar owners (69.7%), construction workers (66.7%), bar attendants (66.1%) and fishermen [(61.0%), p<0.01]. Daily alcohol consumption was highest among older participants (35-49years) and sex workers [p<0.01]. Alcohol consumption before sex (43.0%) was highest among youths (25-34 years), sex workers, bar owners and attendants [p<0.01]. HIV prevalence was higher amongst alcohol consumers [63.4% vs. 36.6%, p<0.01], with daily and weekly consumers having a higher prevalence [73.0% vs. 27.0%, p=0.05].

Illicit drugs like cocaine, Miraa/Khat were used by 13.8% of participants, mostly amongst sex workers [(33%), p<0.01]. Illicit drugs were used mainly on a daily basis [(44.6%), p<0.01].

## Conclusion

Alcohol and illicit drug use is prevalent in FC and associated with higher prevalence of HIV infection. Efforts towards controlling alcohol and illicit drug use might help control HIV in these communities

